# Supplementary material for: Long-range phase coherence and tunable second order φ0-Josephson effect in a Dirac semimetal 1T-PtTe2
Source: Commun Phys. 2024 Oct 28;7(1):354. doi: 10.1038/s42005-024-01825-0 (PMC11519005; doi:10.1038/s42005-024-01825-0)
Supplement: Supplementary file 2 — Supplementary Information [file 42005_2024_1825_MOESM2_ESM.pdf]

Supplementary Information

Long-range Phase Coherence and Tunable Second Order φ_0-Josephson Effect in a Dirac Semimetal 1T-$PtTe_2$

Pranava K. Sivakumar[1], Mostofa T. Ahari[2], Jae-Keun Kim[1], Yufeng Wu[1], Anvesh Dixit[1], George J. de Coster[3], Avanindra K. Pandeya[1], Matthew J. Gilbert[2,4] and Stuart S. P. Parkin[1].

1. Max Planck Institute of Microstructure Physics, 06120 Halle (Saale), Germany
2. Materials Research Laboratory, The Grainger College of Engineering, University of Illinois, Urbana-Champaign, Illinois 61801, USA
3. DEVCOM Army Research Laboratory, 2800 Powder Mill Rd, Adelphi, Maryland 20783, USA
4. Department of Electrical Engineering, University of Illinois, Urbana-Champaign, Illinois 61801, USA

Contents for Supplementary Notes

The supplementary information file contains the following material that supports the data analysis and main conclusions of the manuscript.

1. Thickness of the $PtTe_2$ flake
2. Electrical transport properties of $PtTe_2$ in the normal state
3. Self-consistent treatment of the wide Josephson junction
4. Identification and Minimization of JDE induced by self-field effects in wide $PtTe_2$ Josephson junctions
5. Josephson diode effect in a vertical Josephson junction of $PtTe_2$
6. ΔI_c due to the geometric shape inversion asymmetry in $PtTe_2$ junctions
7. Effect of finite thickness of $PtTe_2$ flake on the interference pattern
8. Flux focusing and Estimation of effective junction area for diffraction pattern calculations
9. Evolution of Fraunhofer patterns in positive and negative magnetic fields for L1
10. ΔI_c from device L3
11. ΔI_c from device L4
12. List of all measured junctions with different seperations
13. Effect of magnetic flux on ΔI_c
14. Accidental SQUID in junction L2
15. Josephson diode efficiency in $PtTe_2$ junctions
16. Discussion on g-factor estimation from the JDE

Supplementary References

Supplementary Note 1: Thickness of the $PtTe_2$ flake

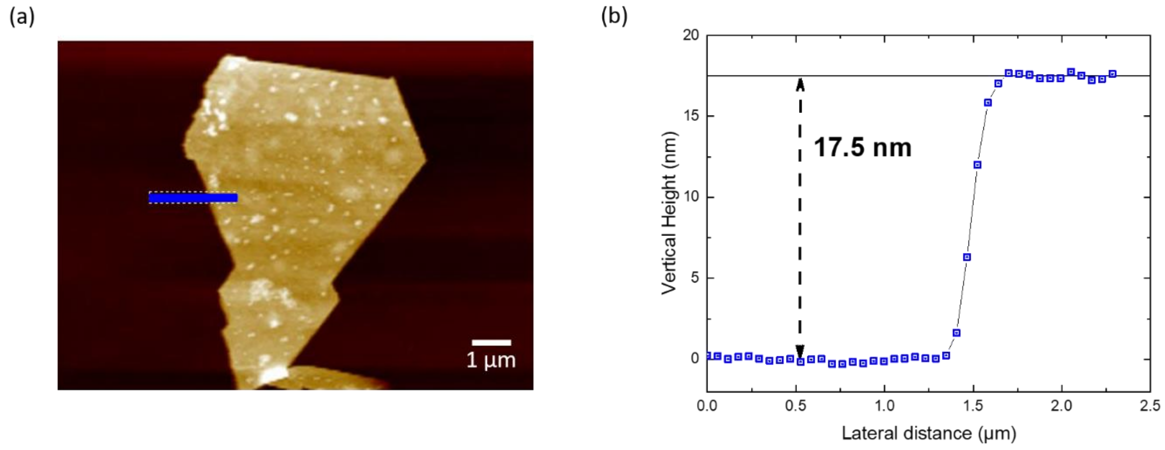

Fig. S1 AFM image and thickness of $PtTe_2$ flake used. (a) AFM image of the as exfoliated $PtTe_2$ flake on Si/SiO_2 substrate. The scale bar corresponds to 1 µm. (b) shows the height of the $PtTe_2$ flake measured from AFM measurement at the position marked with a blue line in (a).

Supplementary Note 2: Electrical transport properties of $PtTe_2$ in the normal state

A separate Hall bar device with Ti (2 nm) / Au (40 nm) contacts was fabricated on a 30 nm thick $PtTe_2$ flake using a similar method as described above to measure its electrical properties in the normal state as shown in Fig. S2. Electrical measurements are performed using a current source (Keithley 6221) with a constant current bias of 10 µA and the voltage measured using a nanovoltmeter (Keithley 2182A). It is found that $PtTe_2$ remains metallic down to 20 mK temperature and does not turn superconducting. It can also be seen from the positive magnetoresistance and linear Hall effect that $PtTe_2$ is non-magnetic down to the base temperature and hence time-reversal symmetric. The mobility of the flake as estimated from the magnetoresistance is $\mu_{\mathrm{MR}} = 835\ \mathrm{cm^2V^{-1}s^{-1}}$ and the carrier concentration calculated from the Hall resistance is $n = 1.424 \times 10^{28}\ \mathrm{m^{-3}}$.

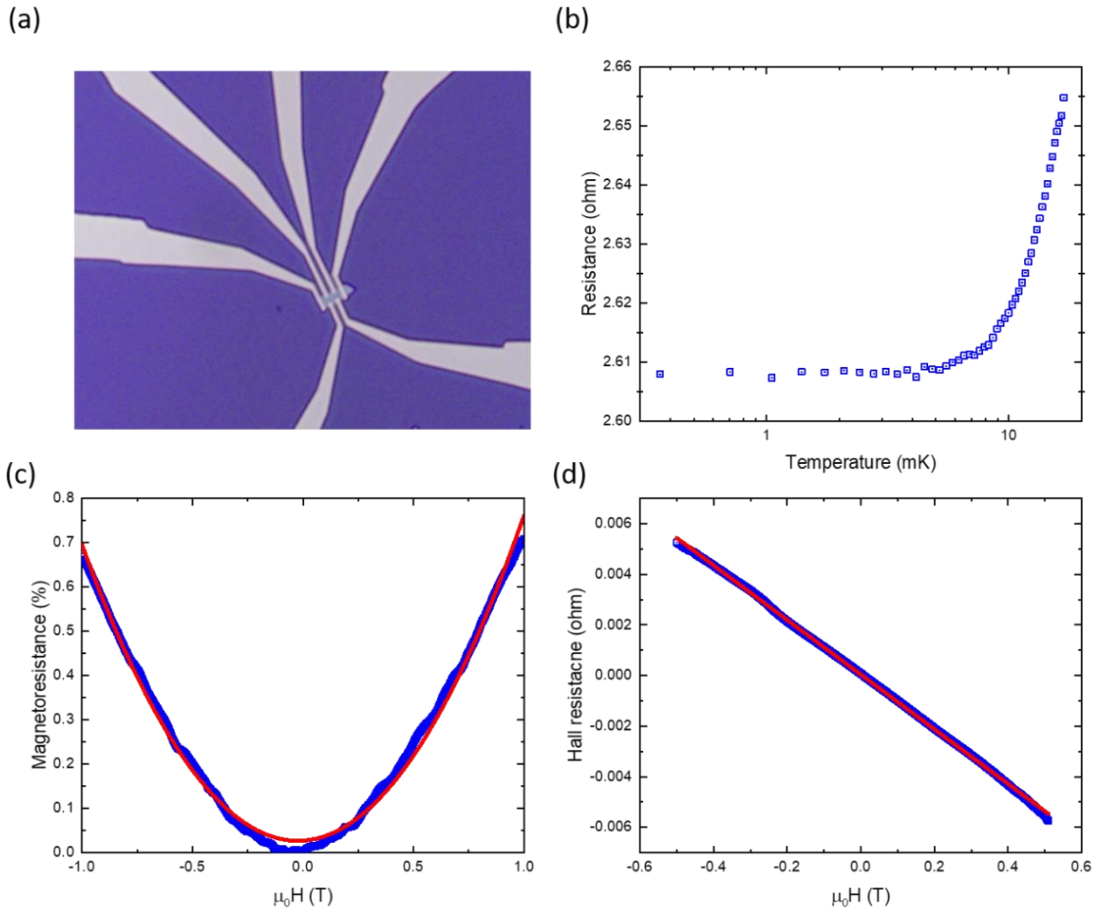

Fig. S2 Electrical transport properties of an exfoliated $PtTe_2$ flake. (a) Optical image of a Hall bar device with gold electrodes fabricated on a $30\ nm$ thick $PtTe_2$ flake exfoliated on Si/SiO_2 substrate. (b) Resistance of the $PtTe_2$ flake measured as a function of temperature shows that it remains metallic down to $20\ mK$. (c) Magnetoresistance of the $PtTe_2$ flake is positive and shows quadratic behavior indicating the absence of magnetism in the system. (d) The Hall resistance of the $PtTe_2$ flake is linear with a negative slope indicating the transport is hole-like with a carrier density of $n = 1.424 \times 10^{28}\ \mathrm{m}^{-3}$.

Supplementary Note 3: Self-consistent treatment of the wide Josephson junction

Consider the Josephson junction under a uniform external magnetic field. Since the junction thickness in the z direction $t \approx 17$ nm is much less than the London penetration depth (λ_{L}), as result, we expect a large self-inductance influencing the phase φ, and B_{y} penetrates the junction without distortion. In the presence of out-of-plane filed B_{z}, the phase φ obeys the differential equation

$$\mu_0 J_{\mathrm{c}} \lambda_{\mathrm{J}}^2 \frac{\partial \varphi}{\partial y} = B_{\mathrm{z}}, \qquad (1)$$

where μ_0 is the permeability of free space, $J_{\mathrm{c}} \equiv J_1$ is the critical density, λ_{J} is the Josephson penetration length. Since λ_{J} depends on the details of the junction [1-3] as we discuss below, we use different current bias configurations to directly obtain λ_{J}. When the current density is sufficiently strong, the supercurrent flow in the junction tends to screen the magnetic field from the interior of the junction[4], and the magnetic field satisfies the Maxwell equation

$$\frac{\partial B_{\mathrm{z}}}{\partial y} = \mu_0 J_{\mathrm{x}}(y) \qquad (2)$$

Now, combining Eqs. (1) and (2), we write

$$\frac{\partial^2 \varphi}{\partial y^2} = \frac{1}{\lambda_{\mathrm{J}}^2}\left(\sin\varphi + \frac{J_2}{J_1}\sin 2\varphi + \delta\right) \qquad (3)$$

We note that when $J_2 = 0$, Eq. (3) reduces to the usual wide junction limit with a sinusoidal CPR, the so-called (static) sine-Gordon equation[5]. However when $J_2 \neq 0$, Eq. (3) is known as the (static) double sine-Gordon equation. Note that in the main text, when we discuss the CPR we use $I_2 \equiv J_2 tW$, and $I_1 \equiv J_1 tW$. The DC limit of our junction is well captured by solutions of this equation, which corroborates the validity of our CPR. Converting to dimensionless equations, we adopt the boundary conditions[6], for the phase gradient as

$$W \frac{\partial \varphi}{\partial y}\bigg|_{y=-\frac{W}{2}} \approx \alpha 2\pi \frac{\Phi}{\Phi_0} + a_1 \left(\frac{W}{\lambda_\mathrm{J}}\right)^2 \frac{I}{I_\mathrm{c}}$$

$$W \frac{\partial \varphi}{\partial y}\bigg|_{y=\frac{W}{2}} \approx \alpha 2\pi \frac{\Phi}{\Phi_0} + a_2 \left(\frac{W}{\lambda_\mathrm{J}}\right)^2 \frac{I}{I_\mathrm{c}}$$

where $I_\mathrm{c} = J_\mathrm{c} t W,\ \ \alpha = \Phi_0/(2\pi\mu_0 d_\mathrm{eff} J_\mathrm{c} \lambda_\mathrm{J}^2)$ is a dimensionless constant parameter which we determine from our simulation to be $\alpha \approx 1$, with $d_\mathrm{eff} = d + 2\lambda_\mathrm{L}$, and $I = t\int_{-W/2}^{W/2} J_\mathrm{x}(y)dy$ is the total supercurrent flowing in the junction. Here Φ is the external magnetic field's flux threading the junction. The same-side and opposite-side (criss-crossed) biasing can now be easily adopted by $(a_1, a_2) = (0,1)$ and $(-1/2, 1/2)$, respectively. It is clear that total current affects the solutions of the phase, and, in turn, the phase determines the current density $J_\mathrm{x}(y)$. As a result, these equations are solved self-consistently. In order to obtain the Fraunhofer patterns, we find the maximum and minimum total supercurrents, $I_\mathrm{c}^+ = \max[I]$ and $I_\mathrm{c}^- = \min[I]$, that satisfy the boundary conditions. . At zero in-plane field $B_\mathrm{y} = 0$, Fig. S3c and S3f present our simulated Fraunhofer patterns with $\lambda_\mathrm{J} = W/2$ for same-side and opposite side biasing, respectively. We point out that, depending on the value of $\frac{J_2}{J_1}$, our simulations with the penetration length within the range $1 < W/\lambda_\mathrm{J} < 4$ can match well with the experimental results.

For the opposite-side bias boundary conditions, one can check that a self-consistent (numerical) solution of Eq. (3) produces the main features of Fraunhofer patterns, such as lifted nodes and a local minimum (dip) in I_c^-. However, for simplicity we consider a simple solution $\varphi = 2\pi \frac{\Phi}{\Phi_0}\frac{y}{W} + \frac{1}{2}\left(\frac{W}{\lambda_\mathrm{J}}\right)^2 \frac{I}{I_\mathrm{c}} \left|\frac{y-y_0}{W}\right|$ that satisfies the boundary conditions and captures the central local minimum feature in the Fraunhofer patterns. While our simulations in the main text are for $y_0 = 0$, a nonzero y_0 can lead to an asymmetry in Fraunhofer patterns as $I_\mathrm{c}^\pm(B) \neq I_\mathrm{c}^\pm(-B)$.

Supplementary Note 4: Identification and Minimization of JDE induced by self-field effects in wide $PtTe_2$ Josephson junctions

In a Josephson junction, when the junction width (W) is smaller than the Josephson penetration depth (λ_J), which is a characteristic length scale in the junction over which magnetic flux variation can take place, the magnetic field due to the current flow through the junction can be neglected. Such a junction is said to be in the 'short junction limit'. In contrast when the width of the junction is larger in comparison to the Josephson penetration depth $(W > \lambda_J)$, the effect of current-induced magnetic field becomes significant. In such limit, the geometry of the current source configuration can play a significant role in the current distribution across the junction. In a lateral junction as here, when the current source is connected to leads on the same side of the device and the voltage probes are connected to the leads on the other side (as in Fig. S3a), this can lead to a non-uniform current distribution in the junction. This consequently creates local inhomogeneous magnetic fields in the junction that can break the time reversal symmetry of the junction. This is known in the literature as a 'self-field effect (SFE)'[7-9] and is dictated purely by the geometry of the junction and the magnitude of the critical current. While measuring the current-phase relationship of the junction by applying a magnetic field perpendicular to the plane of the sample (B_z), this SFE can lead to the creation of Fraunhofer oscillations that are skewed to one side and which alternates with the current direction. Such a skewed Fraunhofer pattern has been depicted in Fig. S3c. It is clear that there is a difference in the critical currents that is induced by skewing of this interference pattern, which leads to a ΔI_c as shown in the inset to Fig. S3c. This ΔI_c induced by SFE looks very similar to those induced by finite momentum Cooper pairing in the system[10] but arises purely due to the extrinsic SFE and has little to do with the intrinsic properties of the junction material. Hence, it is important to make sure that the skewedness and JDE arising due to such geometric effects are completely nullified while measuring ΔI_c and deriving conclusions about the material properties based on it. We note that even though the SFE evidently breaks time-reversal symmetry upon application of a current, there is no spontaneous time-reversal symmetry breaking in the absence of a current and the inhomogeneous magnetic fields induced by the currents in positive and negative directions are the same in magnitude. Hence, there is no ΔI_c at zero magnetic field in the presence of SFE-inducing currents.

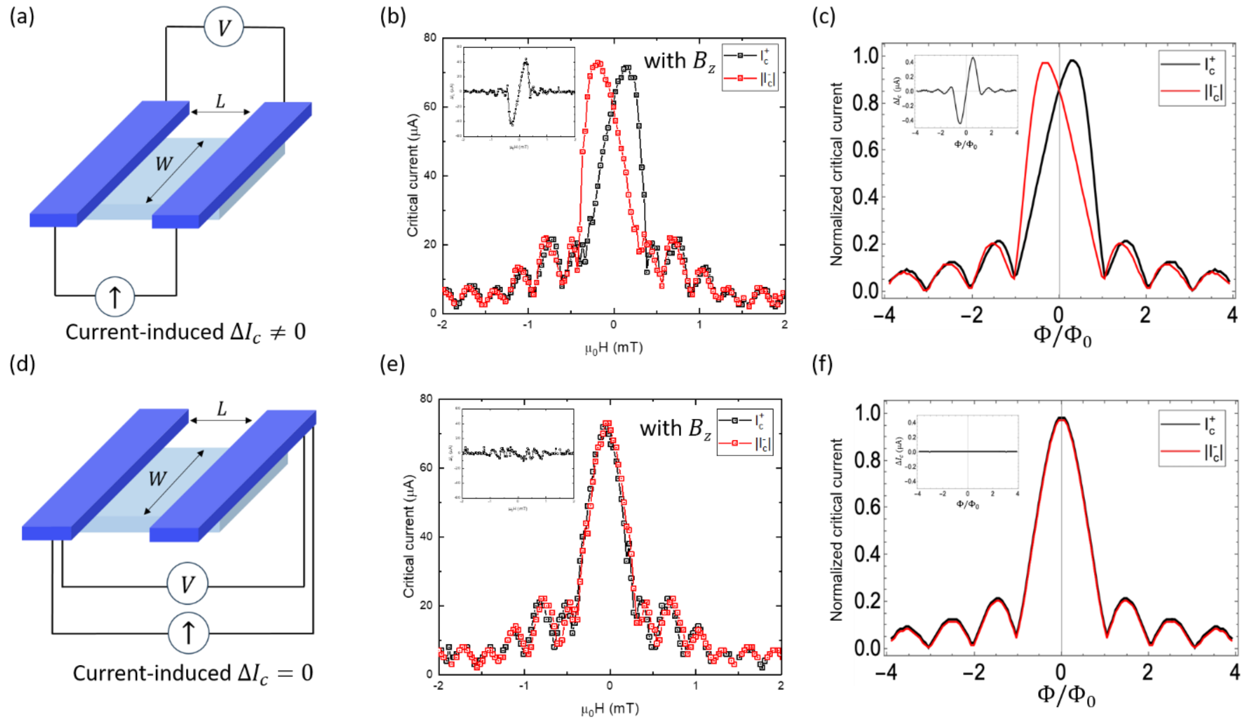

Fig. S3 Self-field effects in wide $PtTe_2$ junctions. (a) Measurement schematic for a wide lateral Josephson junction in which self-field effects were observed in the Fraunhofer pattern. (b) Skewed Fraunhofer pattern with large ΔI_c (inset) measured with the current leads on the same side. (c) Simulated Fraunhofer patterns for same-side biasing with $W \sim \lambda_J$. The self-field effects gives rise to skewed Fraunhofer patterns as in experiments. The calculated ΔI_c are shown in the inset. (d) Measurement schematic in which the current bias is sourced in the "criss-crossed" configuration, wherein the current leads are located on opposite sides of the superconducting electrodes. (e) The Fraunhofer pattern for I_c^+ and I_c^- measured in the criss-crossed configuration is symmetric with respect to the magnetic field with negligible ΔI_c (inset) indicating the near uniform flow of supercurrents. (f) Simulated Fraunhofer patterns for criss-crossed biasing with $W = 2\lambda_J$. In this case, self-field effects are symmetric and lead to symmetric Fraunhofer patterns where $\Delta I_c = 0$ (inset). In (c) and (f), we plot the normalized critical currents as a function of normalized magnetic flux Φ/Φ_0.

In L1, the junction channel is between $5 - 6$ μm wide while the estimated λ_J for the junction is $\lambda_J \approx 3$ μm for $B_y = 0$, which makes L1 lie in the wide junction limit $(W > \lambda_J)$. The

evolution of the critical current in the presence of B_z is first measured in a geometry where the current source is connected to leads located on the same side of the junction as in Fig. S3a. It displays a skewed critical current behavior (Fig. S3b), which is not symmetric with the magnetic field direction and deviates strongly from the expected behavior for Fraunhofer patterns. I_c^+ and I_c^- are found to be skewed along opposite directions leading to a large ΔI_c, as shown in the inset to Fig. S3b. The observed ΔI_c reaches a maximum value of around 40 μA, when the total maximum critical current is only 73 μA with a very large diode efficiency $\left(\eta = \frac{\Delta I_c}{I_c^+ + |I_c^-|}\right)$ of around 47 %. The simulated Fraunhofer pattern for a wide Josephson junction with SFE is also shown for comparison in Fig. S3c. This result shows the presence of a strong SFE and a non-uniform current distribution when the current bias is applied in the geometrical configuration as in Fig. S3a. The ΔI_c observed has a large magnitude and strongly mimics that created by spin-momentum locking, including the oscillatory behavior with magnetic field[10]. If not analyzed carefully, this would lead to the possible conclusion that there is a Zeeman-type out-of-plane spin-momentum locking in the system that leads to a large ΔI_c with a magnetic field along z-axis as in Ising superconductors such as 2H-$NbSe_2$[11]. However, this is not true in our case. To remove the skewedness of critical currents, we use a 'criss-crossed geometry' of the current source. In the criss-crossed configuration (Fig. S3d), for which the current source is connected to the leads on opposite sides of the device, the skewed nature of the Fraunhofer pattern vanishes completely and I_c^+ and I_c^- fall on top of each other, as shown in Fig. S3e with a negligible ΔI_c (in the inset to Fig. S3e). The Fraunhofer pattern displays periodic oscillations of the critical current and is quite symmetric with respect to the direction of magnetic field. This indicates that the distribution of supercurrents, when the current bias is applied in the criss-crossed configuration, is rather uniform and there is negligible extrinsic JDE in the system due to the junction geometry.

Supplementary Note 5: Josephson diode effect in a vertical Josephson junction of $PtTe_2$

A vertical Josephson junction (V1) of a 60 nm thick $PtTe_2$ flake was fabricated using $NbSe_2$ as the superconducting electrode on the top and bottom using a dry transfer technique with a polycarbonate (PC) film coated on a dome-shaped polydimethylsiloxane (PDMS) stamp as described in the literature[12], to look for a Josephson diode effect (ΔI_c) by passing supercurrents along the c-axis (as shown in Fig. S4a). The vertical heterostructure formed is then dropped on pre-sputtered gold electrodes at 200 ℃ and immersed in chloroform to remove any PC residue. The vertical stack is then annealed in vacuum at 300 ℃ for an hour to improve the electrical contact to the flakes. No apparent ΔI_c was observed, when a magnetic field was applied in the plane of the flakes along different directions as shown in Fig. S4b and Fig. S4c even though the Josephson energy and the magnitude of the maximum critical current is quite similar in both the L1 and V1 junctions. This demonstrates that possibly there is no spin-momentum locking in the bulk of the sample and the observation of a ΔI_c in the presence of a magnetic field is confined to current flow along the two-dimensional ab-plane of the sample.

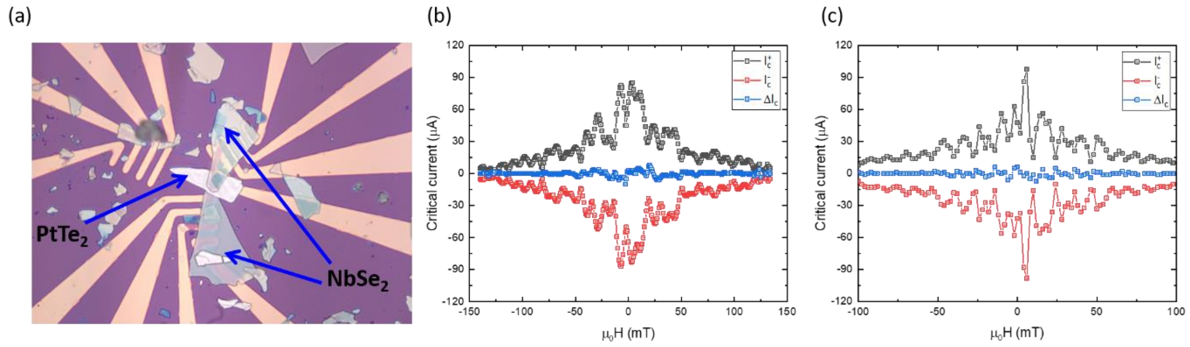

Fig. S4 Absence of Josephson diode effect in a vertical junction of $PtTe_2$. (a) Optical image of a vertical $PtTe_2$ Josephson junction with superconducting $NbSe_2$ electrodes in the top and bottom. (b) and (c) Critical currents I_c^+ (black) and I_c^-(red) of the vertical junction measured as a function of the in-plane magnetic field at 20 mK with magnetic field applied along two perpendicular directions show that ΔI_c (blue) is almost zero and has no apparent trend.

Supplementary Note 6: ΔI_c due to the geometric shape inversion asymmetry in $PtTe_2$ junctions

The trapezoidal shape and tapering edges of the $PtTe_2$ flake in Josephson junctions L1-L4 naturally break the inversion symmetry of the system and might be considered as the origin for the observed ΔI_c in these junctions. If this is indeed the case, then junctions L1 and L3, which taper in opposite directions should show opposite signs of ΔI_c when the current and magnetic field are applied along the same direction in both these devices. We show below that this is not the case and we observe ΔI_c for L1 and L3 is of the same sign when measured under the same conditions, hence ruling out the likelihood of geometric asymmetry contributing significantly to the observed ΔI_c.

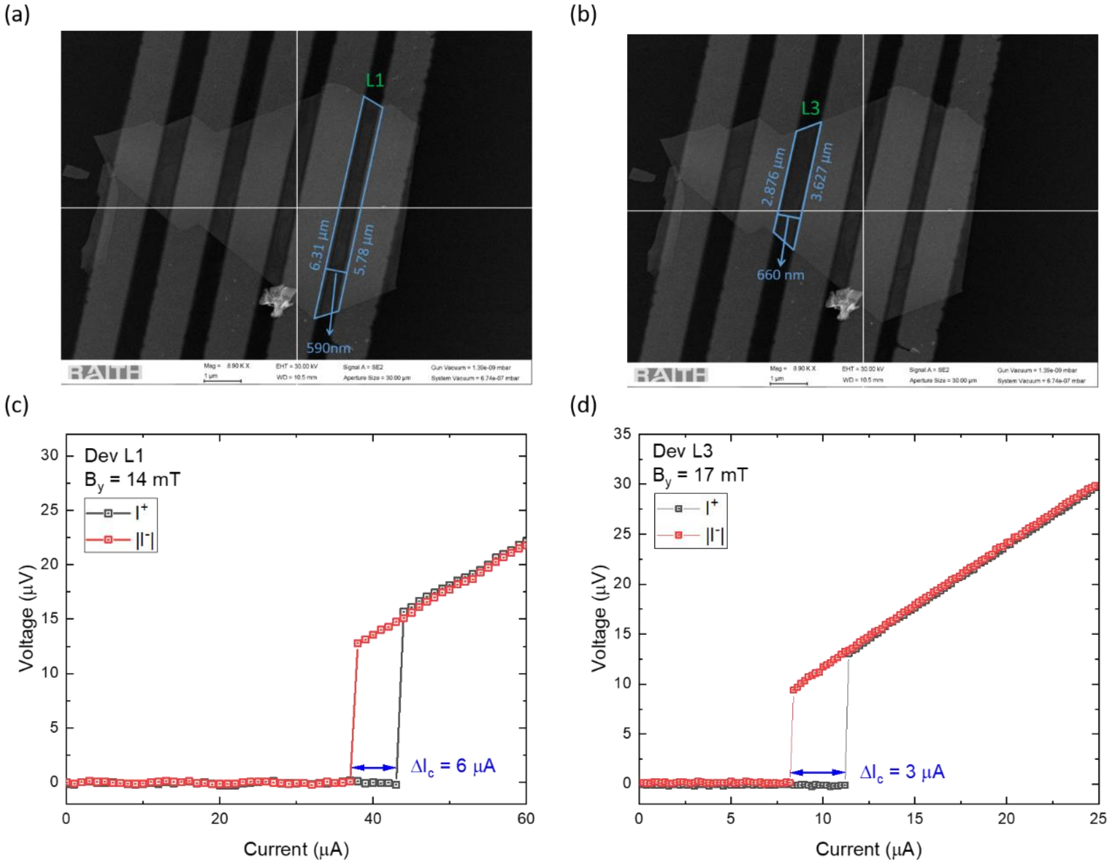

Fig. S5 Josephson diode effect in junctions of different geometry. (a) and (b) Scanning electron microscope (SEM) image of lateral $PtTe_2$ Josephson junctions L1 and L3 (described in the main text) with an outline of their trapezoidal shapes and a measurement of their dimensions. (c) and

(d) ΔI_c for L1 and L3 measured for a positive B_y with the same direction of current bias show that both these junctions have the same sign of ΔI_c under these conditions.

Supplementary Note 7: Effect of finite thickness of $PtTe_2$ flake on the interference pattern

When the critical current of the junction is measured as a function of the magnetic flux along the z-axis (B_z) which induces a phase difference between the superconducting electrodes, we see the expected Fraunhofer interference pattern. When the Fraunhofer pattern is mapped as a function of different in-plane magnetic fields along the y-axis (B_y), we observe that there is a uniform shift of the whole Fraunhofer pattern along the B_z with increasing B_y, which can be identified by tracking the position of the central maxima. This shift can be effectively modeled by replacing B_z with $B_z + \gamma B_y$, where γ is a small fitting parameter introduced when B_y deviates from the y-direction. This shift has also been observed previously as a tilt of the entire Fraunhofer map in similar measurements used to estimate finite-momentum of the Cooper pairs in Josephson junctions[10,13,14]. This tilt is then corrected by subtracting a linear slope that brings the central Fraunhofer maxima back to zero B_z.

In our measurements to track the evolution of I_c^+ and I_c^- in the Fraunhofer oscillations with B_y, we employ a similar procedure to correct for the observed shift of the Fraunhofer pattern by fixing the position of the central maxima at $B_z = 0\ mT$. Below we show the Fraunhofer patterns as measured at different values of B_y (Fig. S6a-d) and after performing the shift correction (Fig. S6e-h) for junction L1. When B_y is increased beyond 30 mT it becomes hard to track the central peak, so the slope of the peak shift with B_y at lower values of B_y can be used to do the shift correction. All Fraunhofer pattern analyses presented in the main text are done after performing this correction.

We note that, despite the correction via a uniform shift, the Fraunhofer patterns are observed to be asymmetric with respect to Φ. We believe this asymmetry is due to the geometric asymmetry in the width of the two leads[14], i.e., W_1 and W_2 (the trapezoidal shape of the junction).

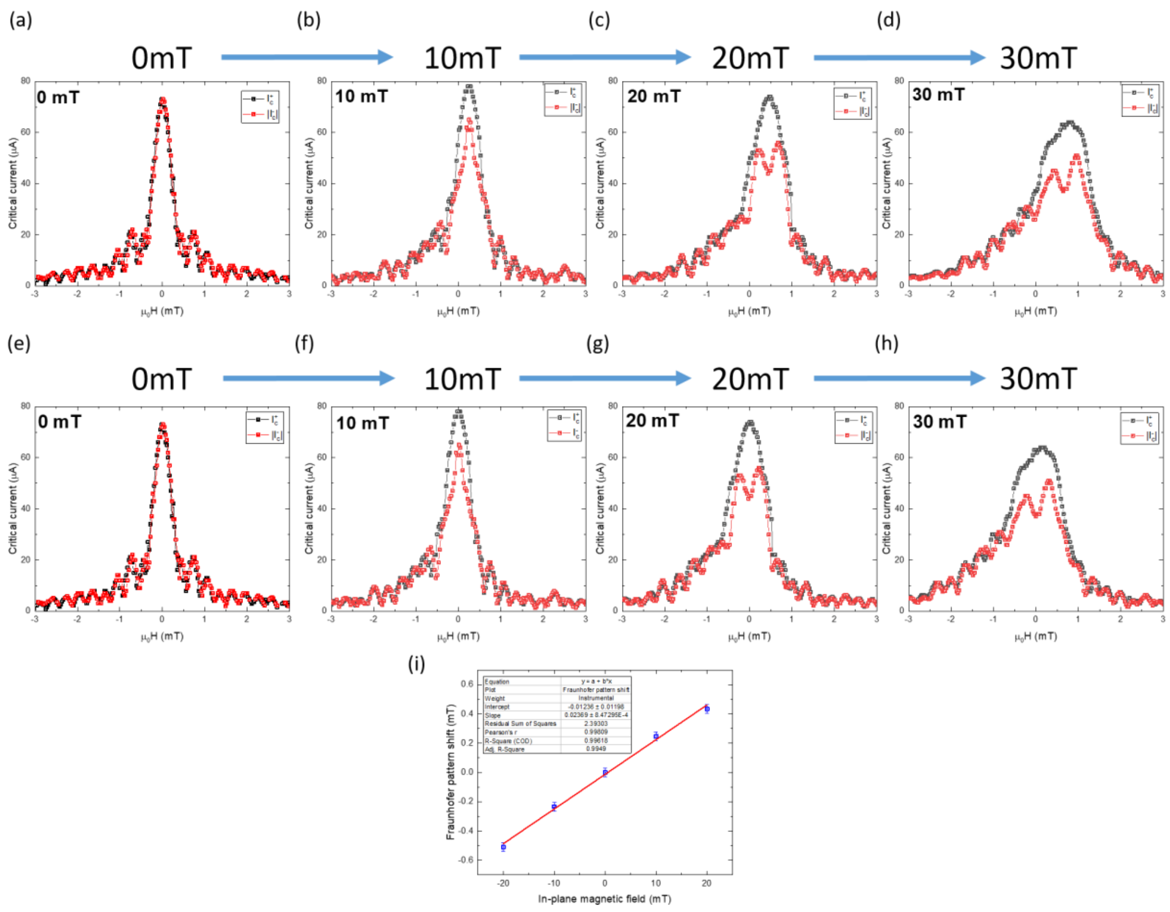

Fig. S6 Evolution of Fraunhofer pattern for L1 under an in-plane magnetic field and shift correction. (a)-(d) The Fraunhofer patterns for both I_c^+ and I_c^- are found to shift towards the right side along positive B_z values for increasing positive values of B_y and similarly along negative B_z values for negative values of B_y. (e)-(h) The Fraunhofer patterns for different positive B_y values after performing the shift correction setting the central maxima of I_c^+ to be at $B_z = 0$ mT. (i) Plot showing the linear shift of the Fraunhofer pattern with an in-plane magnetic field B_y.

Supplementary Note 8: Flux focusing and Estimation of effective junction area for diffraction pattern calculations

In order to properly estimate the period of oscillations in the observed diffraction pattern, it is important to precisely calculate the effective area of the junction through which the Josephson current flows and the effective magnetic flux through the junction. First, the effective area of the junction including the London penetration depth is calculated and then the effective flux through the junction including flux focusing effects is calculated.

Since the junctions on the $PtTe_2$ flake are shaped like a trapezoid, which is a regular quadrilateral, it makes the calculation of the effective junction area easier. While calculating the area of the junction, it is important to take into account the London penetration depth (λ) of the niobium electrodes that can increase the effective separation between the two superconducting electrodes. The London penetration depth for thin films of niobium can vary from 37 nm, which is the bulk value up to 200 nm for different thicknesses and temperatures[15-17]. We use a λ value of ~ 100 nm as reported in literature[15] for films of thicknesses used in our junctions. That would make the effective junction separation to be $d_{\text{eff}} = (2\lambda + d)$. Then the area of the junction is calculated by using the formula for area of a trapezium $A = \left(\frac{a+b}{2}\right).d_{\text{eff}}$, where a and b are lengths of the edges of the junction along the electrodes. Using the values estimated from the SEM image in Fig. S5a, this would give an effective junction area of around $3.5665\ \mu\text{m}^2$ for L1. If we use this as the area of the junction, the magnetic flux through the junction can be calculated as $\Phi = B_z.A$ and this magnetic flux normalized to the magnetic flux quantum $\left(\Phi_0 = \frac{h}{2e}\right)$ would be $\left(\frac{\Phi}{\Phi_0}\right)$. The Fraunhofer pattern in these units in presented in Fig. S8a. As it can be observed, the period of oscillations seem to be around $\left(\frac{\Phi_0}{2}\right)$. Ideally, this would only be possible in the case where the second harmonic term in the current-phase relationship (CPR) is the dominant term and the first harmonic component is virtually non-existent in the junction. The expected Fraunhofer patterns for various ratios of the second and first-harmonic components $\left(\frac{I_2}{I_1}\right)$ is presented in Fig.S7. It can be seen that in order to obtain prominent second harmonic oscillations, as we do in our measurements the second-order term (I_2) needs to much larger than the first-order term (I_1). However, we argue that this is not the case in our junctions as the second-order term stems as a

perturbation only because of high transparency in the junction and can't be larger than the first-order term. We also argue that the observed period is due to the effect of flux focusing that is very well known to occur in lateral Josephson junctions.[18,19]

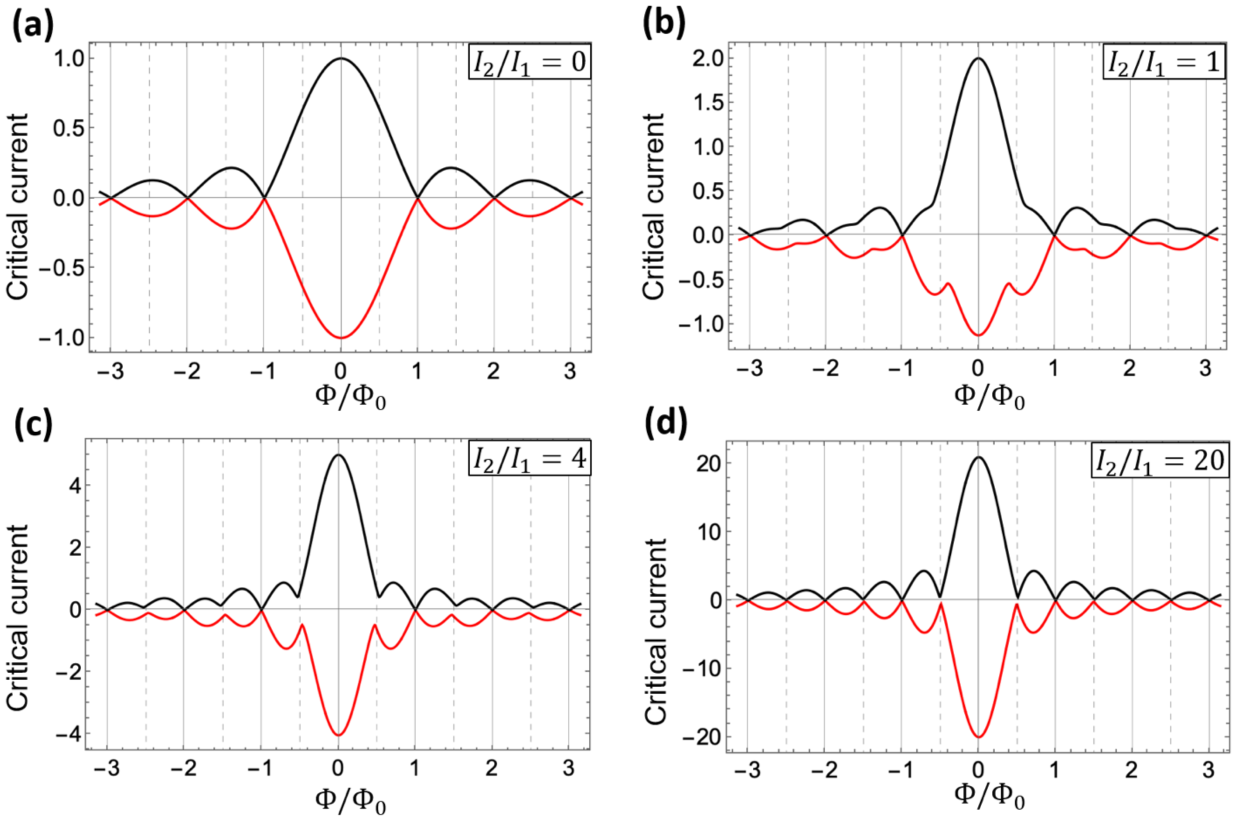

Fig. S7 The calculated Fraunhofer interference pattern for various values of $\mathbf{I_2/I_1}$. (a) The simulated Fraunhofer patterns expected for various values of I_2/I_1 with $\delta = 0$. It can be seen that in order to get prominent $\left(\frac{\Phi_0}{2}\right)$ −periodic oscillations as we observe in our measurements, the ratio of I_2/I_1 needs to be very large.

When a magnetic field is applied to a lateral Josephson junction, the Meissner screening currents in the superconducting electrodes deflect a portion of the magnetic flux towards the junction that results in an increased effective magnetic flux than that expected. This is known as the flux focusing effect, which modifies the expected spacing of nodes in Fraunhofer pattern from Φ to some $\Gamma\Phi$, where the flux scaling factor Γ is given by[18]:

$$\Gamma = \frac{n\Phi_0}{B_z^{(n)} LW},$$

where $L = 590$ nm, $W \approx 6$ µm are the junction length and width (for junction L1). $B_z^{(n)}$ is the out of the plane magnetic field at node n. A uniform spacing of the nodes are observed as the niobium electrodes are deep in the superconducting state at 20 mK and the Meissner screening effects are constant over the scanned range of B_z. For $n = 1$, we have $B_z^{(n)} = 0.3$ mT, which gives $\Gamma \approx 1.9$ that accounts for the observed $\sim\left(\frac{\Phi_0}{2}\right)$ period of the oscillation in the measurements. The Fraunhofer pattern after flux focusing correction is presented in Fig. S8b. The period of oscillations matches well with the expected Φ_0 after accounting for flux focusing effects.

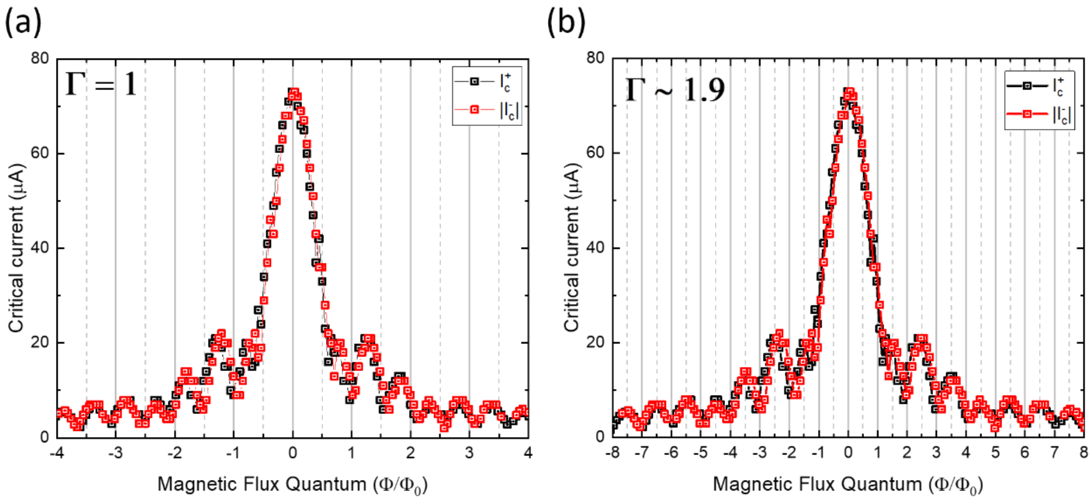

Fig. S8 The Fraunhofer interference pattern for L1 under before and after correcting for flux focusing. (a) The as-plotted Fraunhofer patterns for both I_c^+ and I_c^- are found to have a $\left(\frac{\Phi_0}{2}\right)$ period before flux focusing correction ($\Gamma = 1$). (b) The Fraunhofer pattern after correcting the applied magnetic flux with the calculated flux scaling factor ($\Gamma \sim 1.9$) matches well with the expected Φ_0 period.

Supplementary Note 9: Evolution of Fraunhofer patterns in positive and negative magnetic fields for L1

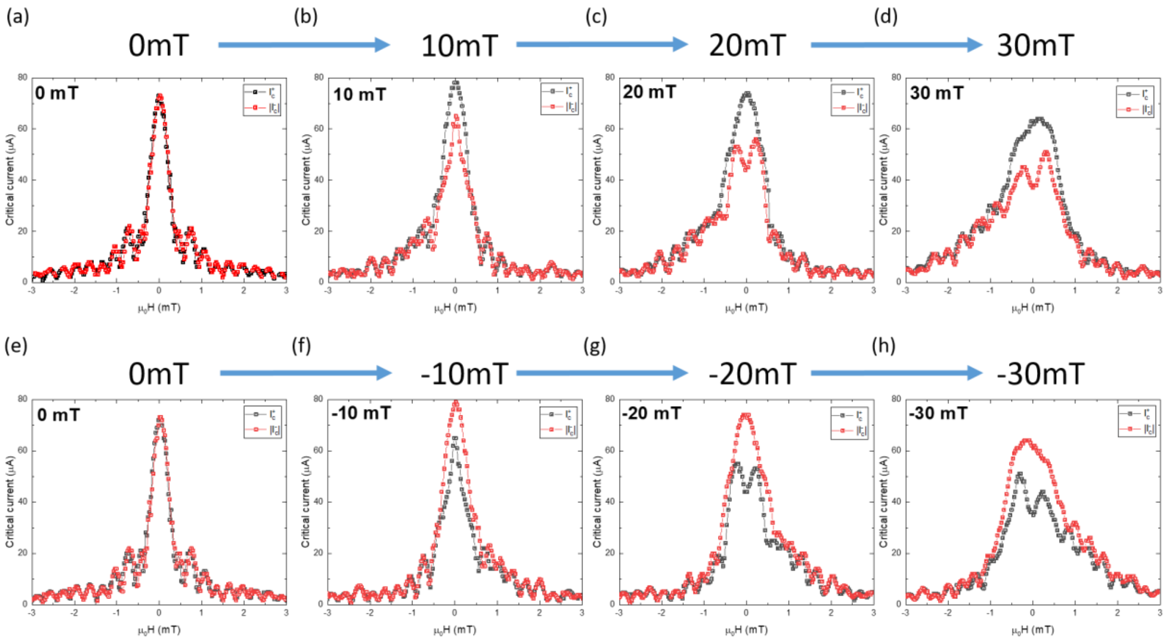

Fig. S9 The Fraunhofer interference pattern for L1 under positive and negative magnetic fields after shift correction. (a)-(d) The Fraunhofer patterns for I_c^+ and I_c^- measured in the presence of a positive B_y magnetic field as shown in the main text. (e)-(h) The Fraunhofer pattern for I_c^+ and I_c^- measured in the presence of a negative B_y magnetic field. The behavior of I_c^+ and I_c^- are reversed under opposite B_y but maintain the symmetry $I_c^{\pm}\left(B_y, B_z\right) = I_c^{\mp}\left(-B_y, -B_z\right)$.

Supplementary Note 10: ΔI_c from device L3

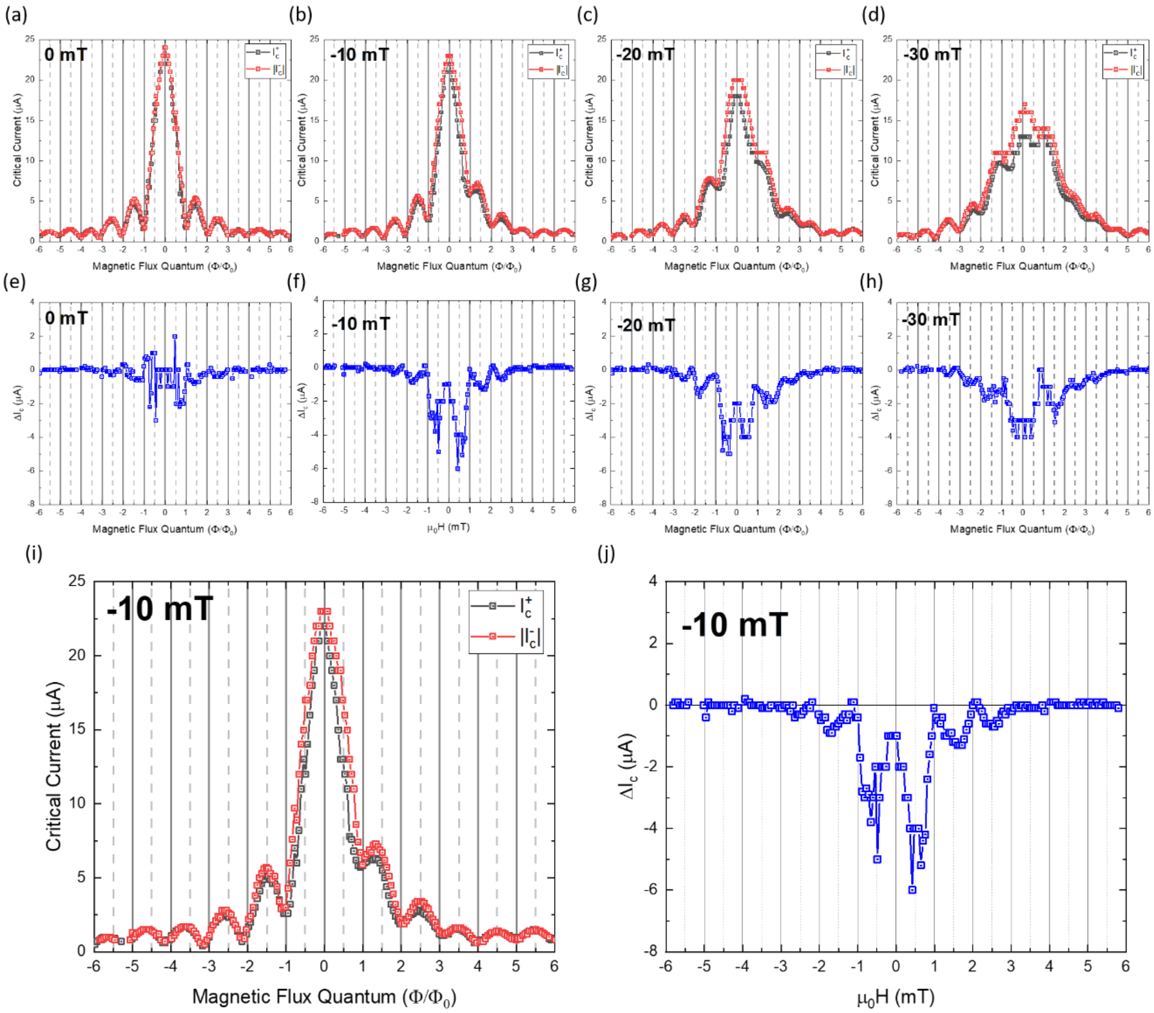

Fig. S11 Evolution of the Fraunhofer pattern in the presence of ΔI_c for L3. (a)-(d) The Fraunhofer patterns for both I_c^+ and I_c^- for L3 with increasing B_y. (e)-(h) Corresponding ΔI_c for the Fraunhofer patterns. ΔI_c for L3 is much smaller compared to L1 as the critical current is also smaller but the $\left(\frac{\Phi_0}{2}\right)$ period of the oscillations can still be distinguished. (i),(j) Larger image of the -10 mT data with only a couple of oscillations visible in ΔI_c.

Supplementary Note 11: ΔI_c from device L4

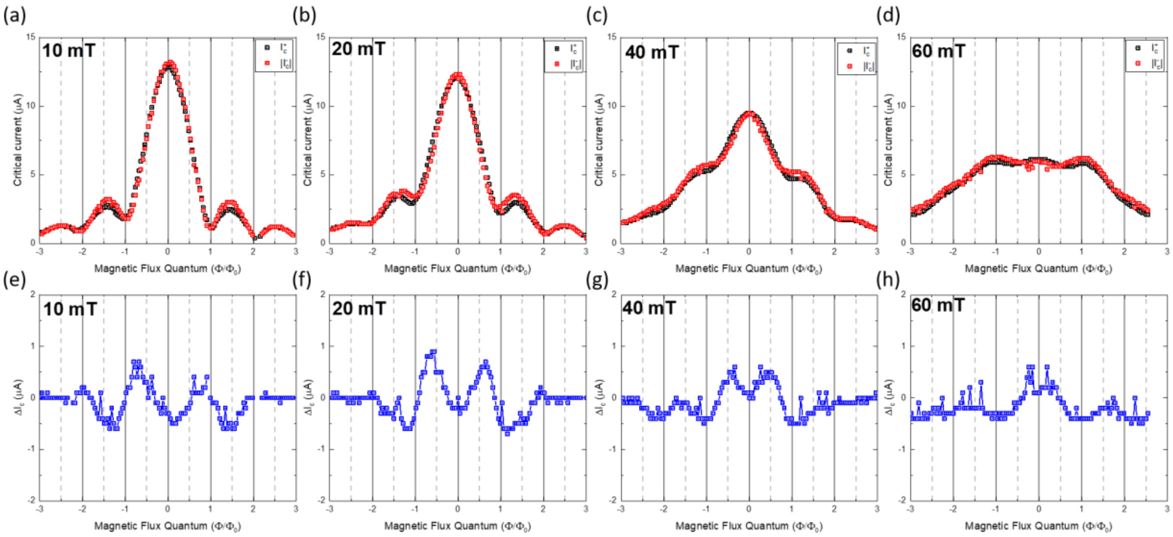

Fig. S12 Evolution of the Fraunhofer pattern in the presence of ΔI_c for L4. (a)-(d) The Fraunhofer patterns for both I_c^+ and I_c^- for L4 with increasing B_y. (e)-(h) Corresponding ΔI_c for the Fraunhofer patterns. ΔI_c in this case is very small and the oscillations are barely discernible.

Supplementary Note 12: List of all measured junctions with different separations

$PtTe_2$ device	Separation d (nm)	Critical current I_c (μA)	R_N (Ω)	I_cR_N (μV)	Second harmonic I_2 (μA)	Transparency τ
L1	390	73	0.37	27.156	19.7	0.45 (from I_e)
L3	466	24	1.2	28.8	1.898	0.435
L4	597	13.4	1.72	23.04	0.52	0.427

Table S1 Table of parameters for all measured junctions. This table contains the critical currents, resistances, transparencies and extracted second harmonic components from the JDE for all junctions on the $PtTe_2$ flake.

Supplementary Note 13: Effect of magnetic flux on ΔI_c

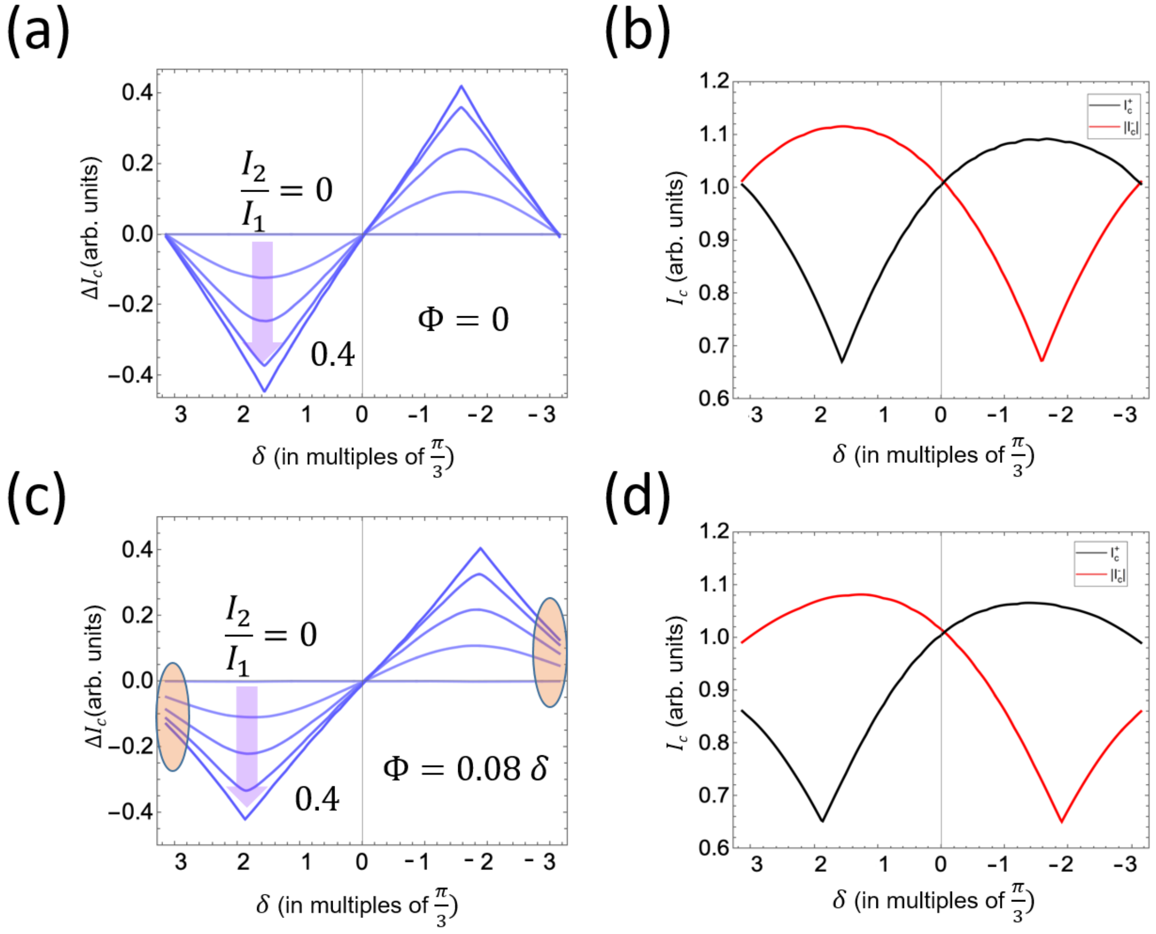

Fig. S13 Evolution of ΔI_c in the presence of a small magnetic flux (Φ). (a), (b) show the simulated behavior of ΔI_c, I_c^+ and I_c^- using the CPR in equation (3) of the main text. (c),(d) show the simulated behavior of ΔI_c in the presence of a magnetic flux (Φ) inside the sample that can modify the behavior and lead to a shift in the position of the nodes in ΔI_c expected from the CPR. The figures are simulated with an additional flux of $\Phi = 0.08\,\delta$, $\delta \rightarrow 0.8\,\delta$ and $\beta = 3$. The circled areas display the lifted nodes. The evolution of ΔI_c shown in (c) with a shift in the position of the node in ΔI_c from occurring at $\delta = \pm\pi$ is consistent with the experimental observation in Fig. 4c, indicating the presence of a small flux resulting from the application of B_y.

Supplementary Note 14: Accidental SQUID in junction L2

(a) **(b)**

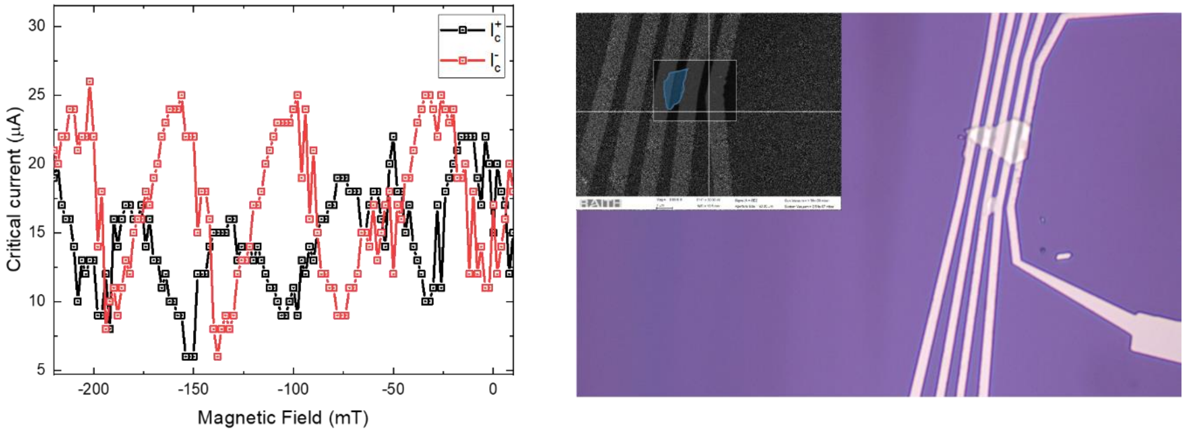

Fig. S14 Accidental SQUID in junction L2. Junction L2 is shorted by another flake of $PtTe_2$ by accident forming an asymmetric SQUID loop. (a) The asymmetric SQUID also shows non-reciprocal critical currents with B_y and highly skewed non-sinusoidal oscillations providing further evidence of the presence of higher harmonics in the CPR. (b) Optical image of the shorted junction. Inset shows a close-up SEM image of the flake shorting L2.

Supplementary Note 15: Josephson diode efficiency in $PtTe_2$ junctions

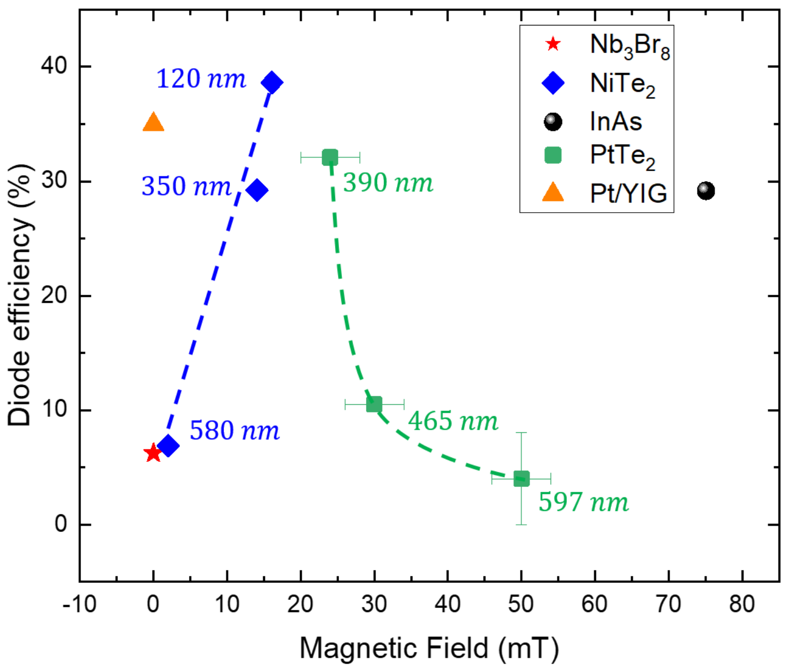

Fig. S15 Evolution of Josephson diode efficiency in $PtTe_2$. The efficiency for various Josephson junction based supercurrent diodes are presented[10,20-22], including the current work calculated using the formula $\eta = \frac{\Delta I_c}{I_c^+ + |I_c^-|}$ is presented. The efficiency of ΔI_c in $PtTe_2$ goes up drastically with decreasing separation and increasing the width of the $PtTe_2$ junctions.

Supplementary Note 16: Discussion on *g*-factor estimation from the JDE

An accurate theoretical estimate of the g-factor requires first-principles calculations of the band structure, which would account for both orbital and multiband contributions to the g-factor. For the purpose of this discussion, assuming the Fermi velocity of the bands contributing to superconductivity is $v_F \approx 3.3 \times 10^5$ ms^{-1} from literature[23], we can estimate the g-factor of the

electrons in $PtTe_2$ from the Cooper pair momentum. The Cooper pair momentum due to the Zeeman effect is given by: $2q = \frac{2g\mu B_y}{\hbar v_F}$ [10,13,14] The diode effect is maximized when $\delta = \frac{\pi}{2}$, that is $2q. d_{eff} = \frac{\pi}{2}$, which in case of device B1 occurs when $B_y = 24mT$. This gives the Cooper pair momentum to be: $2q \approx 2.345 \times 10^6\ m^{-1}$ when $B_y = 24mT$. From this, the value of $\left(\frac{g}{v_\mathrm{F}}\right)$ can be estimated to be around $6.3 \times 10^{-4}\ m^{-1}s$. This estimate indicates that the g-factor is around 208, which is a rather large number and one of the largest values reported to date. However, it should be noted that this estimate depends on the assumed value of v_F and the accurate determination of $d_{eff} = d + 2\lambda$ for the junction, since $g = \frac{\hbar v_\mathrm{F}\pi}{4d_{eff}\mu B_y}$.

In our junctions, the thickness of the superconducting leads is comparable to the coherence length of niobium, which means that λ in our superconducting wire geometry can be significantly larger than that of the λ of thin film niobium reported in literature. In our calculation of $\left(\frac{g}{v_\mathrm{F}}\right)$, we have assumed $\lambda = 100$ nm from literature. Therefore, $\left(\frac{g}{v_\mathrm{F}}\right) \approx 6.3 \times 10^{-4}\ m^{-1}s$ represents rather an upper limit estimate, for the assumed v_F. If we were to assume, for instance, λ of the superconducting electrodes to be around 500 nm instead, this would result in a g-factor of 88. Hence, there is a very large uncertainty in the estimation of g-factor from this method, which depends on the accurate estimation of λ, which in turn depends strongly on various parameters like the exact thickness, geometry and method of deposition of the superconducting electrodes. The estimation of the exact value of λ is further complicated by flux focusing effects in the junction.

Though we cannot precisely estimate the g-factor in this material from the JDE, we would like to note that there have been recent experiments that have demonstrated the existence of extremely large spin-orbit coupling effects in $PtTe_2$. It has been shown to possess a very large spin Hall angle and spin-orbit torques that are much larger than other topological semimetals like WTe_2, Bi_2Se_3 comparable to metallic Pt[24,25] and has also been used in magnetic switching of ferromagnets[26]. While the presence of large spin-orbit coupling in $PtTe_2$ is clear, how its band structure correlates with its unusually large g-factor is something that needs detailed investigation in the future.

Supplementary References

1 Tolpygo, S. K. & Gurvitch, M. Critical currents and Josephson penetration depth in planar thin-film high-Tc Josephson junctions. *Applied Physics Letters* **69**, 3914-3916 (1996).

2 Monaco, R., Koshelets, V. P., Mukhortova, A. & Mygind, J. Self-field effects in window-type Josephson tunnel junctions. *Superconductor Science and Technology* **26**, 055021 (2013). https://doi.org:10.1088/0953-2048/26/5/055021

3 Li, T., Gallop, J. C., Hao, L. & Romans, E. J. Josephson penetration depth in coplanar junctions based on 2D materials. *Journal of Applied Physics* **126**, 173901 (2019). https://doi.org:10.1063/1.5124391

4 Tinkham, M. *Introduction to superconductivity*. (Courier Corporation, 2004).

5 Ferrell, R. A. & Prange, R. E. Self-Field Limiting of Josephson Tunneling of Superconducting Electron Pairs. *Physical Review Letters* **10**, 479-481 (1963). https://doi.org:10.1103/PhysRevLett.10.479

6 Tafuri, F. *Fundamentals and frontiers of the Josephson effect*. Vol. 286 (Springer Nature, 2019).

7 Krasnov, V. M. Josephson junctions in a local inhomogeneous magnetic field. *Phys. Rev. B* **101** (2020). https://doi.org:10.1103/PhysRevB.101.144507

8 Krasnov, V. M., Oboznov, V. A. & Pedersen, N. F. Fluxon dynamics in long Josephson junctions in the presence of a temperature gradient or spatial nonuniformity. *Phys. Rev. B* **55**, 14486-14498 (1997). https://doi.org:10.1103/PhysRevB.55.14486

9 Barone, A. *Physics and Applications of the Josephson Effect*. (Wiley, 2006).

10 Pal, B. *et al.* Josephson diode effect from Cooper pair momentum in a topological semimetal. *Nature Physics* (2022). https://doi.org:10.1038/s41567-022-01699-5

11 Bauriedl, L. *et al.* Supercurrent diode effect and magnetochiral anisotropy in few-layer $NbSe_2$. *Nat. Commun.* **13**, 4266 (2022). https://doi.org:10.1038/s41467-022-31954-5

12 Wang, L. *et al.* One-Dimensional Electrical Contact to a Two-Dimensional Material. *Science* **342**, 614-617 (2013). https://doi.org:doi:10.1126/science.1244358

13 Hart, S. *et al.* Controlled finite momentum pairing and spatially varying order parameter in proximitized HgTe quantum wells. *Nature Physics* **13**, 87-93 (2016). https://doi.org:10.1038/nphys3877

14 Chen, A. Q. *et al.* Finite momentum Cooper pairing in three-dimensional topological insulator Josephson junctions. *Nat Commun* **9**, 3478 (2018). https://doi.org:10.1038/s41467-018-05993-w

15 Gubin, A. I., Il'in, K. S., Vitusevich, S. A., Siegel, M. & Klein, N. Dependence of magnetic penetration depth on the thickness of superconducting Nb thin films. *Phys. Rev. B* **72** (2005). https://doi.org:10.1103/PhysRevB.72.064503

16 Hsu, J. W. & Kapitulnik, A. Superconducting transition, fluctuation, and vortex motion in a two-dimensional single-crystal Nb film. *Phys. Rev. B* **45**, 4819-4835 (1992). https://doi.org:10.1103/physrevb.45.4819

17 Gauzzi, A. *et al.* Very high resolution measurement of the penetration depth of superconductors by a novel single-coil inductance technique. *Rev. Sci. Instrum.* **71**, 2147-2153 (2000). https://doi.org:10.1063/1.1150597

18 Suominen, H. J. *et al.* Anomalous Fraunhofer interference in epitaxial superconductor-semiconductor Josephson junctions. *Phys. Rev. B* **95** (2017). https://doi.org:10.1103/PhysRevB.95.035307

19 Paajaste, J. *et al.* Pb/InAs nanowire josephson junction with high critical current and magnetic flux focusing. *Nano. Lett.* **15**, 1803-1808 (2015). https://doi.org:10.1021/nl504544s

20 Wu, H. *et al.* The field-free Josephson diode in a van der Waals heterostructure. *Nature* **604**, 653-656 (2022). https://doi.org:10.1038/s41586-022-04504-8

21 Jeon, K. R. *et al.* Zero-field polarity-reversible Josephson supercurrent diodes enabled by a proximity-magnetized Pt barrier. *Nat. Mater.* **21**, 1008-1013 (2022). https://doi.org:10.1038/s41563-022-01300-7

22 Baumgartner, C. *et al.* Supercurrent rectification and magnetochiral effects in symmetric Josephson junctions. *Nat. Nanotechnol.* **17**, 39-44 (2022).

23 Amit, Singh, R. K., Wadehra, N., Chakraverty, S. & Singh, Y. Type-II Dirac semimetal candidates ATe_2 (A=Pt, Pd): A de Haas-van Alphen study. *Phys. Rev. Materials* **2**, 114202 (2018). https://doi.org:10.1103/PhysRevMaterials.2.114202

24 Xu, H. *et al.* High Spin Hall Conductivity in Large-Area Type-II Dirac Semimetal $PtTe_2$. *Adv. Mater.* **32**, 2000513 (2020). https://doi.org:https://doi.org/10.1002/adma.202000513

25 Xie, W. *et al.* Wafer-scale synthesis of 2D $PtTe_2$ thin films with high spin–orbit torque efficiency. *Results in Physics* **60**, 107630 (2024). https://doi.org:https://doi.org/10.1016/j.rinp.2024.107630

26 Wang, F. *et al.* Field-free switching of perpendicular magnetization by two-dimensional $PtTe_2/WTe_2$ van der Waals heterostructures with high spin Hall conductivity. *Nat. Mater.* **23**, 768-774 (2024). https://doi.org:10.1038/s41563-023-01774-z
